# Differential roles of exogenous protein disulfide isomerase A3 on proliferating cell and neuroblast numbers in the normal and ischemic gerbils

**DOI:** 10.1002/brb3.1534

**Published:** 2020-01-20

**Authors:** Dae Young Yoo, Su Bin Cho, Hyo Young Jung, Woosuk Kim, Sung Min Nam, Jong Whi Kim, Seung Myung Moon, Yeo Sung Yoon, Dae Won Kim, Soo Young Choi, In Koo Hwang

**Affiliations:** ^1^ Department of Anatomy and Cell Biology College of Veterinary Medicine, and Research Institute for Veterinary Science Seoul National University Seoul South Korea; ^2^ Department of Anatomy College of Medicine Soonchunhyang University Cheonan South Korea; ^3^ Department of Biomedical Sciences, and Research Institute for Bioscience and Biotechnology Hallym University Chuncheon South Korea; ^4^ Department of Anatomy College of Veterinary Medicine Konkuk University Seoul South Korea; ^5^ Department of Neurosurgery Dongtan Sacred Heart Hospital College of Medicine Hallym University Hwaseong South Korea; ^6^ Research Institute for Complementary & Alternative Medicine Hallym University Chuncheon South Korea; ^7^ Department of Biochemistry and Molecular Biology Research Institute of Oral Sciences College of Dentistry Gangneung‐Wonju National University Gangneung South Korea

**Keywords:** cell proliferation, dentate gyrus, differentiated neuroblasts, protein disulfide isomerase A3, transient forebrain ischemia

## Abstract

**Introduction:**

We examined the effects of exogenous protein disulfide isomerase A3 (PDIA3) on hippocampal neurogenesis in gerbils under control and ischemic damage.

**Methods:**

To facilitate the delivery of PDIA3 to the brain, we constructed Tat‐PDIA3 protein and administered vehicle (10% glycerol) or Tat‐PDIA3 protein once a day for 28 days. On day 24 of vehicle or Tat‐PDIA3 treatment, ischemia was transiently induced by occlusion of both common carotid arteries for 5 min.

**Results:**

Administration of Tat‐PDIA3 significantly reduced ischemia‐induced spontaneous motor activity, and the number of NeuN‐positive nuclei in the Tat‐PDIA3‐treated ischemic group was significantly increased in the CA1 region compared to that in the vehicle‐treated ischemic group. Ki67‐ and DCX‐immunoreactive cells were significantly higher in the Tat‐PDIA3‐treated group compared to the vehicle‐treated control group. In vehicle‐ and Tat‐PDIA3‐treated ischemic groups, the number of Ki67‐ and DCX‐immunoreactive cells was significantly higher as compared to those in the vehicle‐ and Tat‐PDIA3‐treated control groups, respectively. In the dentate gyrus, the numbers of Ki67‐immunoreactive cells were comparable between vehicle‐ and Tat‐PDIA3‐treated ischemic groups, while more DCX‐immunoreactive cells were observed in the Tat‐PDIA3‐treated group. Transient forebrain ischemia increased the expression of phosphorylated cAMP‐response element‐binding protein (pCREB) in the dentate gyrus, but the administration of Tat‐PDIA3 robustly increased pCREB‐positive nuclei in the normal gerbils, but not in the ischemic gerbils. Brain‐derived neurotrophic factor (BDNF) mRNA expression was significantly increased in the Tat‐PDIA3‐treated group compared to that in the vehicle‐treated group. Transient forebrain ischemic increased BDNF mRNA levels in both vehicle‐ and Tat‐PDIA3‐treated groups, and there were no significant differences between groups.

**Conclusions:**

These results suggest that Tat‐PDIA3 enhances cell proliferation and neuroblast numbers in the dentate gyrus in normal, but not in ischemic gerbils, by increasing BDNF mRNA and phosphorylation of pCREB.

## Graphical abstract

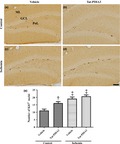

## 1 INTRODUCTION

Cardiac arrest and resuscitation in addition to interruption of blood flow to the brain cause brain tissue damage including neuronal degeneration in the hippocampus, which is the most ischemic sensitive region of the brain (Cui, Shang, Zhang, Jiang, & Jia, 2016). Neuronal damage in the hippocampus impairs the spatial and short‐term memory (Bendel et al., 2005; Von Euler, Bendel, Bueters, Sandin, & Euler, 2006). The mechanisms of neuronal damage include depletion of ATP, excitotoxicity induced by disturbances in calcium homeostasis, and oxidative stress induced by the formation of reactive oxygen species (Ogawa, Kitao, & Hori, 2007; Sanganalmath et al., 2017). There are few available drugs to show the neuroprotective effects specially if administered soon after the ischemic events.

In adult mammals, hippocampal neurogenesis occurs in the subgranular zone (SGZ) of the dentate gyrus (DG) and also in the subventricular zone (SVZ) of the lateral ventricle. Especially, newly generated cells proliferate in the SGZ of DG, differentiate into neuroblasts, and migrate into granule cell layer (GCL) of DG, and they are finally integrated into granule cells in the DG (von Bohlen Und Halbach, 2007; Bruel‐Jungerman, Rampon, & Laroche, 2007). Several lines of evidence demonstrate that modification of adult hippocampal neurogenesis affects the learning and memory formation related to hippocampus (Gould, Beylin, Tanapat, Reeves, & Shors, 1999; Koehl & Abrous, 2011; Leuner, Gould, & Shors, 2006). Transient forebrain ischemia shortly decreases the differentiated neuroblasts at 3 days after ischemia (Pforte, Henrich‐Noack, Baldauf, & Reymann, 2005), but it is substantially and transiently increased later time point after ischemia (Choi et al., 2012; Iwai et al., 2003; Khodanovich et al., 2018). Therefore, induction of endogenous proliferating cells and differentiated neuroblasts at early time points in the brain tissue can be a potential therapeutic approach against neurological disorders including ischemia.

Protein disulfide isomerase (PDI) is an abundant and important housekeeping protein that catalyzes the formation and disruption of disulfide bond between cysteine residues (Gruber, Cemazar, Heras, Martin, & Craik, 2006). Unfolded protein response (UPR) activated by ER stress may modulate repair processes of neuronal damage in the brain by controlling the neurotrophic factor expression including brain‐derived neurotrophic factor (BDNF), and the regenerative potentials of neural stem cells (Castillo et al., 2015; Martínez et al., 2016; Oñate et al., 2016). In addition, BDNF is one of the most important target molecules in phosphorylated cAMP‐response element‐binding protein at Ser133 (pCREB), which is involved in cell proliferation, neuroblast migration and differentiation, and neuronal survival during hippocampal neurogenesis (Lonze & Ginty, 2002). PDI family members are upregulated in various neurological disorders including amyotrophic lateral sclerosis, spinal cord ischemia, Alzheimer's, Huntington's, and Parkinson's diseases (Andreu, Woehlbier, Torres, & Hetz, 2012; Hwang et al., 2005; Nomura, 2004; Tanaka, Uehara, & Nomura, 2000; Truettner, Hu, Liu, Dietrich, & Hu, 2009). PDIA3, also known as ERp57, is an oxidoreductase chaperone and belongs to the PDI family of 20 proteins (Kozlov, Määttänen, Thomas, & Gehring, 2010). Overexpression of PDIA3 protects neurons from cell toxicity induced by methamphetamine in neuroblastoma cell lines (Pendyala, Ninemire, & Fox, 2012) and also from hypoxic brain damage (Kam et al., 2011; Liu et al., 2015; Tanaka et al., 2000), while downregulation of PDIA3 in the nervous system of mice causes severe motor dysfunction and is associated with the loss of neuromuscular synapses (Woehlbier et al., 2016). PDIA3 also contributes to neurite outgrowth by modulating ER calcium release and cytoplasmic dynamics (LeBlanc & Nemere, 2014). Overexpression of PDIA3 in mice facilitates the regeneration of sciatic nerve after mechanical injury (Castillo et al., 2015), while PDIA3 inhibition in mouse keratinocytes decreases their ability of proliferation and migration (Kim, Yoo, Choi, & Yoon, 2015). However, in central nervous system, PDIA3 would not cross the blood–brain barrier to act directly on hippocampus.

Human immunodeficiency virus‐1 (HIV‐1) Tat would be one of best strategy for cellular delivery into central nervous system because they efficiently transport peptide or proteins which have higher molecular weights than their own (Van den Berg & Dowdy, 2011) because the efficient transduction capability has been confirmed using various peptides or proteins against ischemic damage (Jo et al., 2017; Tu et al., 2015; Zhou et al., 2015). In previous studies, we generated Tat‐PDIA3 fusion protein and observed the intracellular delivery of Tat‐PDIA3 fusion protein (Yoo et al., 2017, 2019) as well as the colocalization of PDIA3 with ER tracker (Yoo et al., 2017). In addition, we found the neuroprotective actions of Tat‐PDIA3 and its mechanisms in the gerbil brain ischemia (Yoo et al., 2019) and rabbit spinal cord ischemia (Yoo et al., 2017). Especially, administration of PDIA3 protected neurons from hydrogen peroxide‐induced oxidative stress in HT22 cells and ischemic damage in the gerbil hippocampal CA1 region (Yoo et al., 2019). In addition, Tat‐PDIA3 significantly ameliorated ischemia‐induced changes of UPRs levels after ischemia/reperfusion (Yoo et al., 2019).

Recently, a few studies have reported the regenerative potential of PDIA3 expression in the peripheral nervous system (Castillo et al., 2015) and keratinocytes (Kim et al., 2015), but there are no studies on the effects of PDIA3 on the regenerating capacities in the hippocampus. Therefore, in the present study, we elucidated the effects of exogenous Tat‐PDIA3 on cell proliferation and neuroblast numbers in the gerbil hippocampus after ischemia/reperfusion. In addition, we also examined the possible mechanisms based on the BDNF and pCREB expression in the hippocampus.

## 2 MATERIALS AND METHODS

### 2.1 Construction of expression vectors

A Tat expression vector and Tat‐PDIA3 fusion protein were prepared as shown in previous studies (Yoo et al., 2017, 2019). Polymerase chain reaction (PCR) was conducted to amplify PDIA3 cDNA based on its primer, and it was ligated into the Tat expression vector after subcloning the PCR product into a TA cloning vector. The Tat‐PDIA3 plasmids were expressed in *Escherichia coli* BL21 cells and purified using a Ni^2 +^‐nitrilotriacetic acid Sepharose affinity column and PD‐10 column chromatography (Amersham) described in previous studies. The purified proteins were treated using Detoxi‐Gel™ Endotoxin Removing Gel (Pierce) to remove endotoxins (Yoo et al., 2017, 2019).

### 2.2 Experimental animals

Animals (male gerbils) used in the present study were purchaged from Japan SLC Inc. The handling and care of the animals conformed to the guidelines of current international laws and policies (National Institutes of Health Guide for the Care and Use of Laboratory Animals, Publication No. 85–23, 1985, revised 1996). The Institutional Animal Care and Use Committee of Seoul National University approved the animal procedures (SNU‐160304‐3).

### 2.3 Experimental design

Animals were divided into four groups (*n* = 10 in each group): vehicle (10% glycerol)‐treated control (sham‐operated) group, Tat‐PDIA3‐treated control group, vehicle‐treated ischemic group, and Tat‐PDIA3‐treated ischemic group. At 3 months of age (50–60 g body weight), the animals received intraperitoneal injection of vehicle or Tat‐PDIA3 (3 mg/kg). Vehicle or Tat‐PDIA3 fusion protein was administered to gerbils once a day for 28 days because neuroblasts and immature neurons transiently express doublecortin (DCX) (Brown et al., 2003; Couillard‐Despres et al., 2005).

### 2.4 Induction of transient forebrain ischemia

On day 24 of vehicle or Tat‐PDIA3 treatment, the animals in vehicle‐treated control group, Tat‐PDIA3‐treated control group, vehicle‐treated ischemic group, and Tat‐PDIA3‐treated ischemic groups were anesthetized with a mixture of 2.5% isoflurane (Baxter) in 33% oxygen and 67% nitrous oxide. Transient forebrain ischemia was induced by obstruction of both common carotid arteries with nontraumatic aneurysm clips, as described previously (Yoo et al., 2016). The body temperature was tightly regulated by a thermometric blanker when the animals recovered from anesthesia. Sham operation was conducted with same method without occlusion of both common carotid arteries.

### 2.5 Spontaneous motor activity

To investigate the effects of Tat‐PDIA3 against ischemic damage, spontaneous motor activity was monitored in the gerbils for 60 min before ischemia and one day after ischemia/reperfusion, as described by Yoo et al. (2019). Motor activity was measured by distance traveled during live observations, and the data were reanalyzed with video sequences by two independent observers to ensure objectivity.

### 2.6 Tissue processing and immunohistochemistry

On day 28 of vehicle or Tat‐PDIA3 treatment, animals in the vehicle‐treated control group, Tat‐PDIA3‐treated control group, vehicle‐treated ischemic group, and Tat‐PDIA3‐treated ischemic group (*n* = 5 in each group) were euthanized with 1.5 g/kg urethane (Sigma‐Aldrich). They were then perfused transcardially as mentioned previously (Yoo et al., 2016, 2019). Thirty‐μm tissue sections were obtained with a cryostat (Leica), and hippocampal tissue sections between 1.4 and 2.0 mm posterior to the bregma, in reference to a gerbil atlas (Loskota, Lomax, & Verity, 1974), were used for immunohistochemical staining for anti‐polyhistidine antibody (1:1,000, Santa Cruz Biotechnology), mouse anti‐NeuN antibody (1:1,000; Millipore), rabbit anti‐Ki67 antibody (1:1,000; Abcam), rabbit anti‐DCX antibody (1:200; Abcam), or rabbit anti‐pCREB antibody (pCREB; 1:400; Cell Signaling Technology, Inc.) according to previously described methods (Jung et al., 2019; Yoo et al., 2019).

### 2.7 Quantitative PCR

On day 28 of vehicle or Tat‐PDIA3 treatment, animals in the vehicle‐treated control group, Tat‐PDIA3‐treated control group, vehicle‐treated ischemic group, and Tat‐PDIA3‐treated ischemic group (*n* = 5 in each group) were euthanized with 1.5 g/kg urethane, and the quantitative real‐time PCR was performed as described by Cao et al. (2011). The primers used for real‐time quantitative PCR were as follows: 5′‐ ATGGGTTACACGAAGGAAGG‐3′ (forward) and 5′‐ CCGAACATACGATTGGGTAGT‐3′ (reverse) for BDNF (accession number: NM_012513.3) and 5′‐AGGCCCCTCTGAACCCTAAG‐3′ (forward) and 5′‐CCAGAGGCATACAGGGACAAC‐3′ (reverse) for β‐actin (accession number: EF156276).

### 2.8 Data analysis

Analysis of polyhistidine or DCX immunoreactivity in the molecular layer (ML) of hippocampal DG was performed using an image analysis system and ImageJ software v. 1.50 (National Institutes of Health) with combination of 256’s gray scale and pixels as described in the previous study (Jung et al., 2017). Relative optical density (ROD) was expressed as a percentage of the vehicle‐treated control group (which was set at 100%).

The number of Ki67‐, DCX‐, and pCREB‐immunoreactive cells was assessed by an analysis system (OPTIMAS software version 6.5; CyberMetrics^®^ Corporation; magnification, 100×) as described in a previous study (Jung et al., 2019).

### 2.9 Statistical analysis

Neuro‐regenerative potentials of Tat‐PDIA3 were determined by analyzing the mean differences with two‐way analyses of variance followed by Bonferroni's post hoc test using GraphPad Prism 5.01 software (GraphPad Software, Inc.). Statistical significance was considered when *p* value was below .05.

## 3 RESULTS

### 3.1 Confirmation of Tat‐PDIA3 delivery into the DG

**Figure 1:**
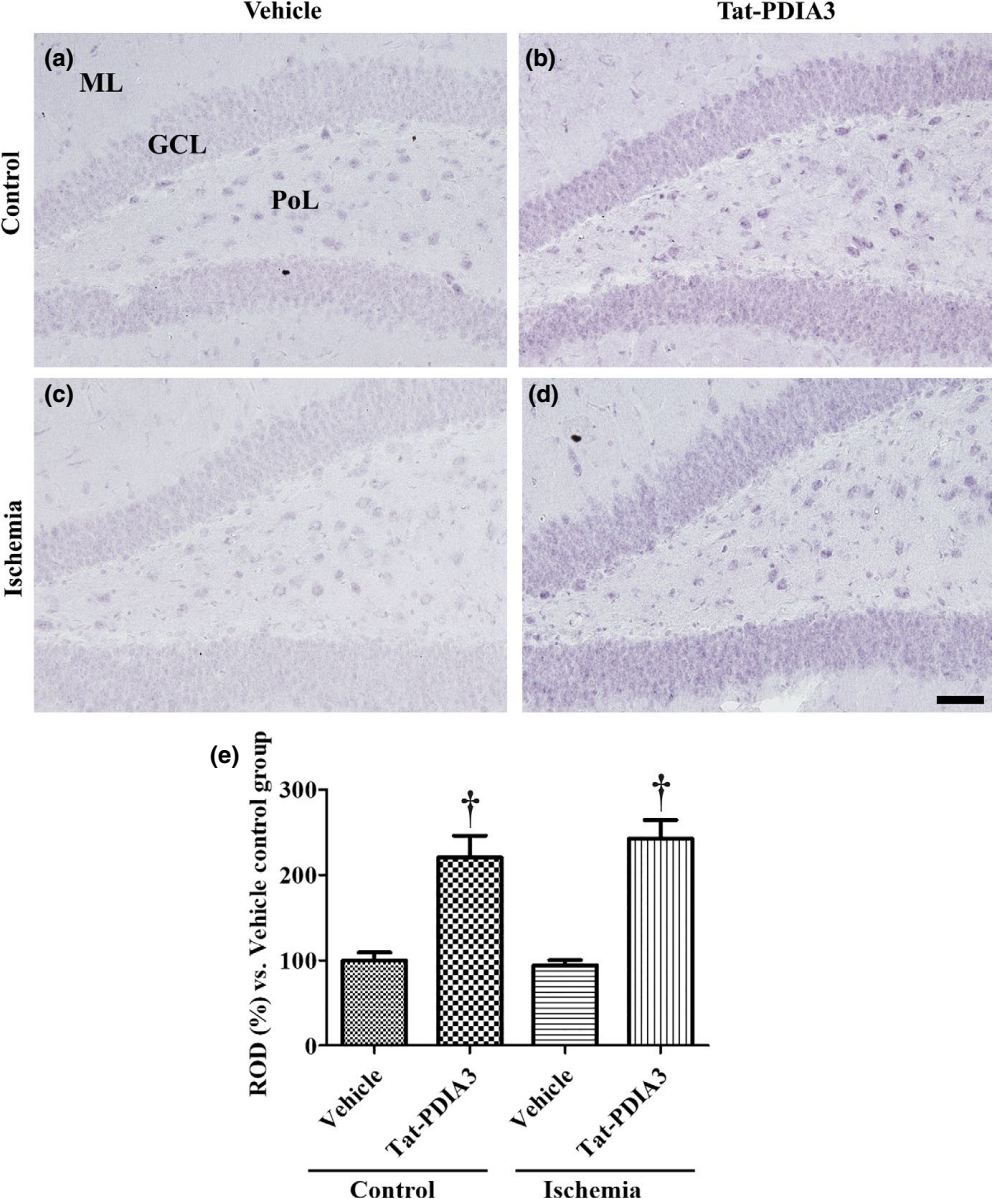
Microphotographs of polyhistidine immunohistochemical staining in the DG of vehicle‐treated control (a), Tat‐PDIA3‐treated control (b), vehicle‐treated ischemic (c), and Tat‐PDIA3‐treated ischemic (d) groups. PoL; polymorphic layer. Scale bar = 100 μm. (e) The relative optical densities (RODs) expressed as a percentage of the value representing the polyhistidine immunoreactivity in the DG of the vehicle‐treated control group are also shown (*n* = 5 per group, ^†^ *p* < .05 indicates a significant difference between vehicle‐ and Tat‐PDIA3‐treated groups). Error bars represent the standard error of the mean

Effective delivery of Tat‐PDIA3 into DG was confirmed by immunohistochemistry for polyhistidine. In vehicle‐treated control group, polyhistidine immunoreactivity was faintly observed in the DG (Figure 1a), while in the Tat‐PDIA3‐treated control group, polyhistidine immunoreactivity strongly observed in all neurons in the DG (Figure 1b) and polyhistidine immunoreactivity was significantly increased in the DG compared to that in the vehicle‐treated control group (Figure 1e). In the vehicle‐treated ischemic group, weak polyhistidine immunoreactivity detected in the DG (Figure 1c) and polyhistidine immunoreactivity was similar to that in the vehicle‐treated control group (Figure 1e). In the Tat‐PDIA3‐treated ischemic group, strong polyhistidine immunoreactivity was found with similar levels compared to that in the Tat‐PDIA3‐treated control group (Figure 1d,e).

### 3.2 Effects of Tat‐PDIA3 fusion protein on spontaneous motor activity in control and ischemic gerbils

**Figure 2:**
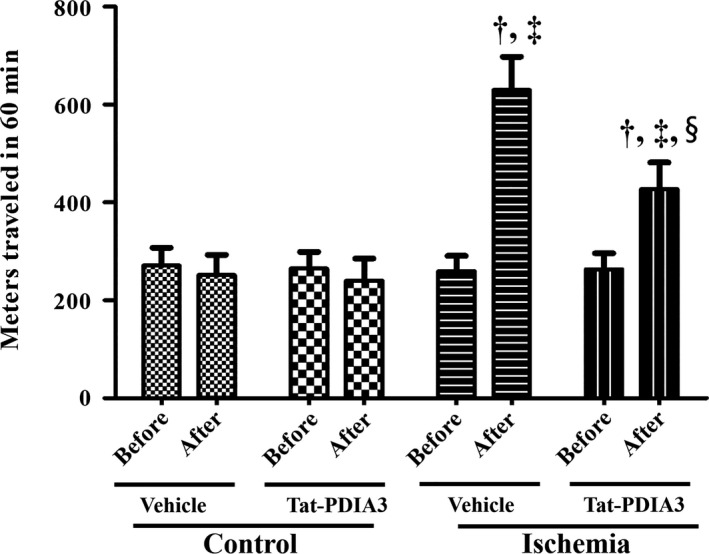
Spontaneous motor activity was measured by traveled distance for 60 min before and one day after ischemia in vehicle‐treated control, Tat‐PDIA3‐treated control, vehicle‐treated ischemic, and Tat‐PDIA3‐treated ischemic groups (*n* = 5 per group; ^†^ *p* < .05 vs. from the same group measured before ischemic surgery, ^‡^ *p* < .05 indicates a significant difference between vehicle‐ and Tat‐PDIA3‐treated groups, and ^§^ *p* < .05 indicates a significant difference between control and ischemic groups). The bars indicate the standard error of the mean

In the vehicle‐treated control and Tat‐PDIA3‐treated control groups, spontaneous motor activity was showed similar levels before and after ischemia. However, in the vehicle‐treated ischemic group, spontaneous motor activity was dramatically increased 1 day after ischemia and the ratio of motor activity before and after ischemia was 2.42 in this group. In the Tat‐PDIA3‐treated ischemic group, spontaneous motor activity was less increased compared to that in the vehicle‐treated ischemic group and the ratio of motor activity before and after ischemia was significantly decreased to 1.62 compared to that in the vehicle‐treated ischemic group (Figure 2).

### 3.3 Effects of Tat‐PDIA3 fusion protein on neuronal death in control and ischemic gerbils

**Figure 3:**
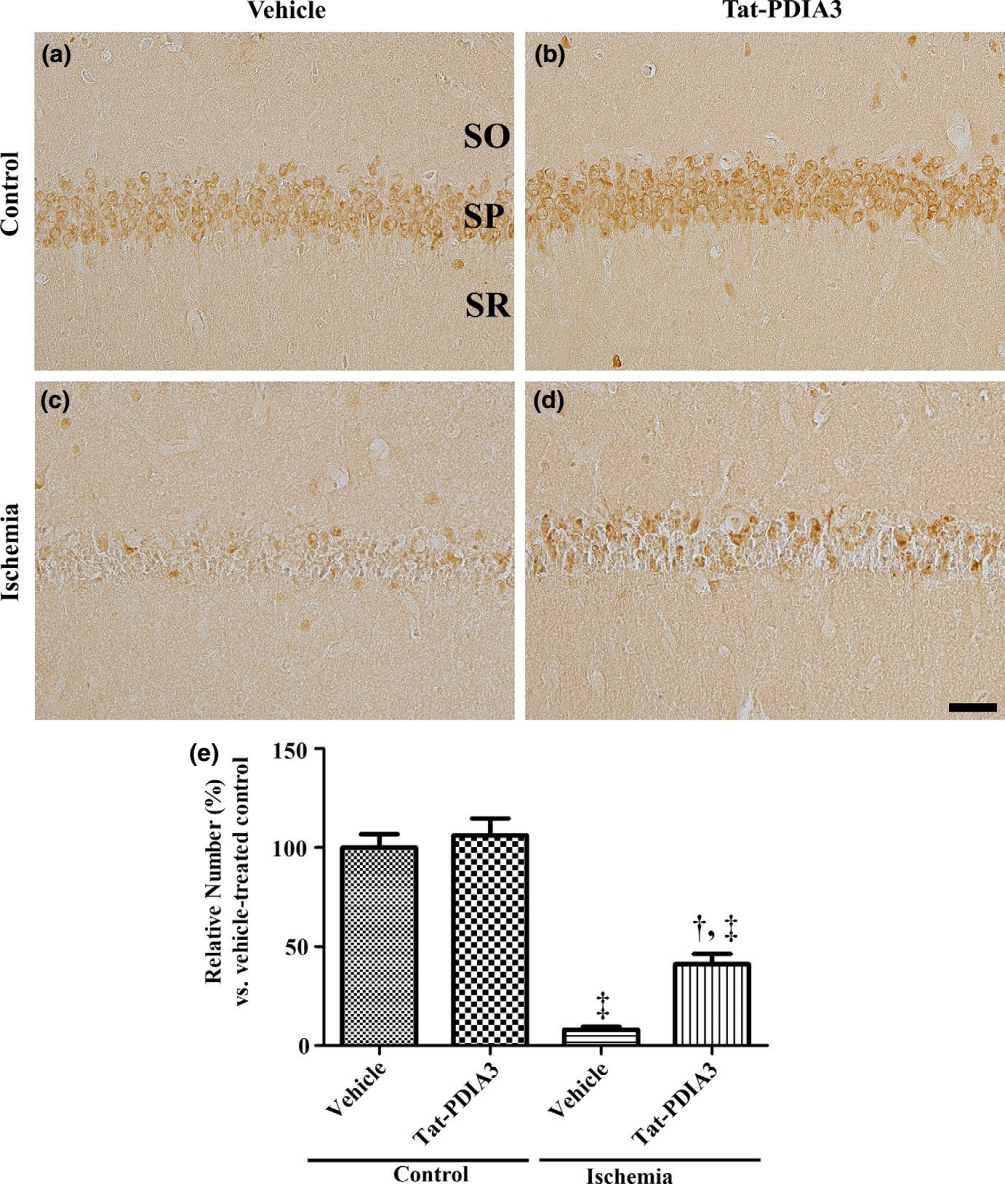
Microphotographs of NeuN immunohistochemical staining in the DG of vehicle‐treated control (a), Tat‐PDIA3‐treated control (b), vehicle‐treated ischemic (c), and Tat‐PDIA3‐treated ischemic (d) groups. PoL; polymorphic layer. Scale bar = 100 μm. (e) The relative number of NeuN‐positive nuclei per section in the DG of each group is shown (*n* = 5 per group, ^†^ *p* < .05 indicates a significant difference between vehicle‐ and Tat‐PDIA3‐treated groups, and ^‡^ *p* < .05 indicates a significant difference between control and ischemic groups). Error bars represent the standard error of the mean

In the vehicle‐treated control and Tat‐PDIA3‐treated control groups, abundant NeuN‐immunoreactive neurons were detected in the hippocampal CA1 region (Figure 3a,b) and there were no significant differences in the number of NeuN‐immunoreactive neurons. In the vehicle‐treated ischemic group, a few NeuN‐immunoreactive neurons were observed in the hippocampal CA1 region (Figure 3c) and the number of NeuN‐immunoreactive neurons was significantly decreased to 8.09% of vehicle‐treated control group (Figure 3e). In the Tat‐PDIA3‐treated ischemic group, some NeuN‐immunoreactive neurons were found in the hippocampal CA1 region (Figure 3d) and the number was significantly increased by 41.19% of vehicle‐treated control group compared to that in the vehicle‐treated ischemic group (Figure 3e).

### 3.4 Effects of Tat‐PDIA3 fusion protein on cell proliferation in control and ischemic gerbils

**Figure 4:**
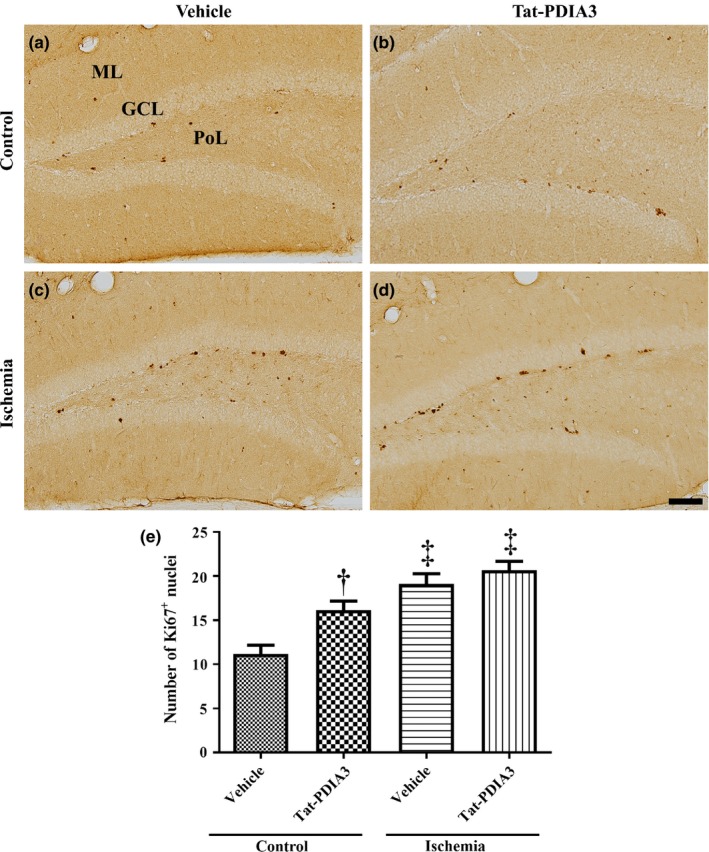
Microphotographs of Ki67 immunohistochemical staining in the DG of vehicle‐treated control (a), Tat‐PDIA3‐treated (b), vehicle‐treated ischemic (c), and Tat‐PDIA3‐treated ischemic (d) groups. PoL; polymorphic layer. Scale bar = 100 μm. (e) The number of Ki67‐positive (^+^) nuclei per section in the DG of each group is shown (*n* = 5 per group, ^†^ *p* < .05 indicates a significant difference between vehicle‐ and Tat‐PDIA3‐treated groups, and ^‡^ *p* < .05 indicates a significant difference between control and ischemic groups). Error bars represent the standard error of the mean

In all groups, Ki67‐positive nuclei were mainly found in the SGZ of the DG. However, the number of Ki67‐positive nuclei was significantly different among the groups. In the vehicle‐treated control group, the number of Ki67‐positive nuclei was 11.2 per sections (Figure 4a,e). In the Tat‐PDIA3‐treated control group, the percentage of Ki67‐positive nuclei was high by 144.3% in comparison with the vehicle‐treated control group (Figure 4b,e). In the vehicle‐ and Tat‐PDIA3‐treated ischemic groups, clustered Ki67‐positive nuclei were detected in the DG and their percentage was 170.7% and 184.6%, respectively, showing that it was not significantly different between these two groups (Figure 4c,d,e).

### 3.5 Effects of Tat‐PDIA3 fusion protein on neuroblast number in control and ischemic gerbils

**Figure 5:**
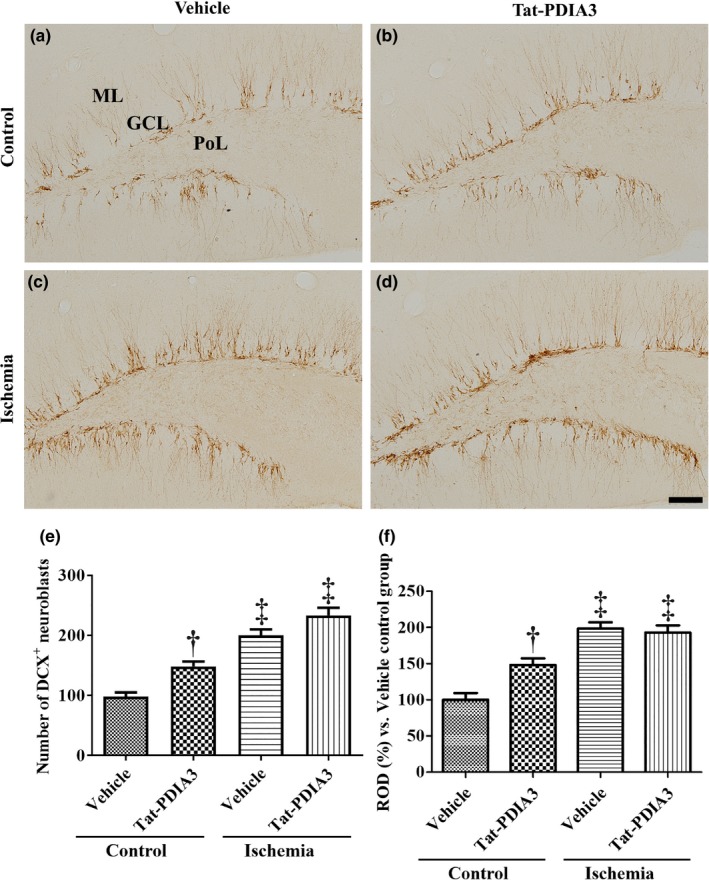
Microphotographs of doublecortin (DCX) immunohistochemical staining in the DG of vehicle‐treated control (a), Tat‐PDIA3‐treated (b), vehicle‐treated ischemic (c), and Tat‐PDIA3‐treated ischemic (d) groups. PoL; polymorphic layer. Scale bar = 100 μm. (e) The number of DCX‐positive (^+^) neuroblasts per section in the DG of each group and (f) the relative optical densities (RODs) expressed as a percentage of the value representing the DCX immunoreactivity in the DG of the vehicle‐treated control group are also shown (*n* = 5 per group, ^†^ *p* < .05 indicates a significant difference between vehicle‐ and Tat‐PDIA3‐treated groups, and ^‡^ *p* < .05 indicates a significant difference between control and ischemic groups). Error bars represent the standard error of the mean

In the vehicle‐treated control group, DCX‐immunoreactive neuroblasts were detected in the SGZ and GCL of the DG and their dendrites extended into the ML of the DG (Figure 5a). In the Tat‐PDIA3‐treated control group, many DCX‐immunoreactive neuroblasts were found in the SGZ of the DG and DCX‐immunoreactive structures are more abundant compared to those in the vehicle‐treated control group (Figure 5b). Especially, the number of DCX‐immunoreactive neuroblasts was 151% higher in the SGZ of DG and DCX immunoreactivity was 148.1% higher in the ML of DG compared to that in the vehicle‐treated control group (Figure 5e,f). In the vehicle‐ and Tat‐PDIA3‐treated ischemic groups, many DCX‐immunoreactive neuroblasts were detected in the GCL of the DG, but some were also found in the SGZ of the DG (Figure 5c,d). The number of DCX‐immunoreactive neuroblasts and DCX immunoreactivity significantly increased in the vehicle‐ and Tat‐PDIA3‐treated ischemic groups compared to those in the vehicle‐ and Tat‐PDIA3‐treated control groups. The number of DCX‐immunoreactive neuroblasts was 204.0% and 238.0%, respectively, in these two groups compared to that in the vehicle‐treated control group, indicating that there was no significant difference in DCX‐positive neuroblast numbers and DCX immunoreactivity between vehicle‐ and Tat‐PDIA3‐treated ischemic groups (Figure 5e,f).

### 3.6 Effects of Tat‐PDIA3 fusion protein on pCREB in control and ischemic gerbils

**Figure 6:**
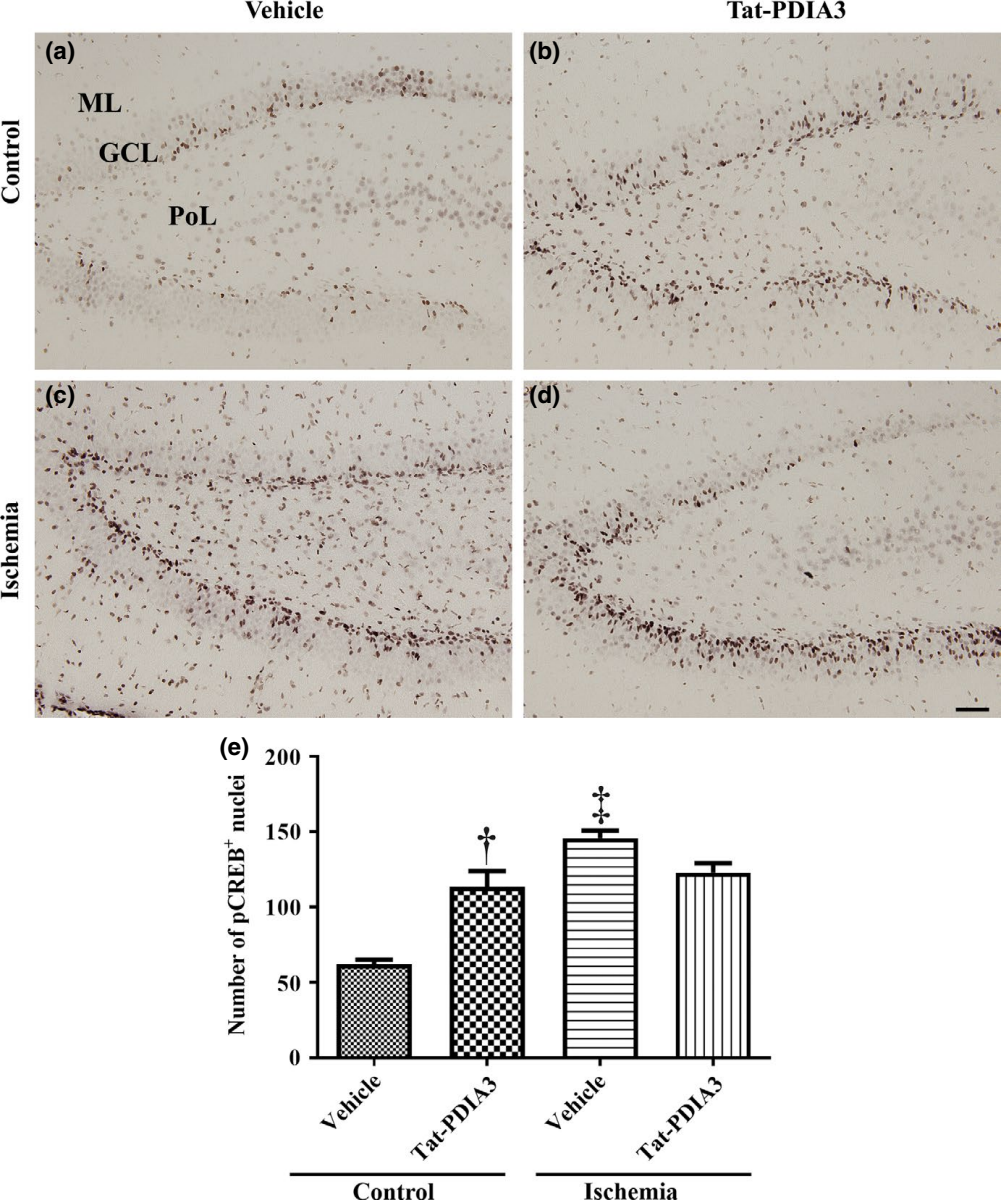
Microphotographs of phosphorylated cAMP‐response element‐binding protein (pCREB) immunohistochemical staining in the DG of vehicle‐treated control (a), Tat‐PDIA3‐treated (b), vehicle‐treated ischemic (c), and Tat‐PDIA3‐treated ischemic (d) groups. PoL; polymorphic layer. Scale bar = 50 μm. (e) The number of pCREB‐positive (^+^) nuclei per section in the DG of each group is shown (*n* = 5 per group, ^†^ *p* < .05 indicates a significant difference between vehicle‐ and Tat‐PDIA3‐treated groups, and ^‡^ *p* < .05 indicates a significant difference between control and ischemic groups). Error bars represent the standard error of the mean

In the vehicle‐treated control group, some pCREB‐positive nuclei were found in the SGZ of the DG and the number of pCREB‐positive nuclei was 62.0 per section (Figure 6a,e). In the Tat‐PDIA3‐treated control group, the percentage of pCREB‐positive nuclei was 183.1% higher compared to those in the vehicle‐treated control group (Figure 6b,e). In the vehicle‐ and Tat‐PDIA3‐treated ischemic groups, pCREB‐positive nuclei were abundantly found in the SGZ and GCL of the DG (Figure 6c,d). The percentage of pCREB‐positive nuclei was 234.9% in the Tat‐PDIA3‐treated ischemic group and 198.1% in the vehicle‐treated ischemic group. There was no significant difference in number of pCREB‐positive nuclei between vehicle‐ and Tat‐PDIA3‐treated ischemic groups (Figure 6e).

### 3.7 Effects of Tat‐PDIA3 fusion protein on BDNF mRNA expression in control and ischemic gerbils

**Figure 7:**
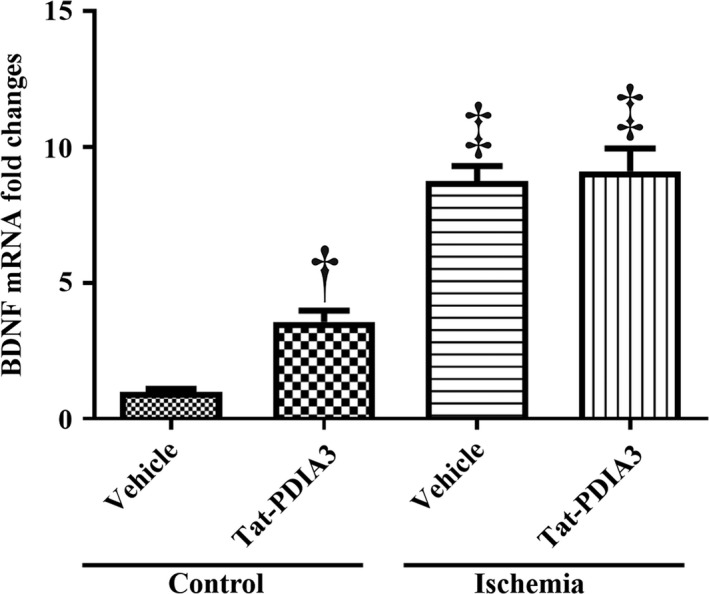
Expression levels of brain‐derived neurotrophic factor (BDNF) mRNA in the hippocampi of vehicle‐treated control, Tat‐PDIA3‐treated, vehicle‐treated ischemic, and Tat‐PDIA3‐treated ischemic groups (*n* = 5 per group, ^†^ *p* < .05 indicates a significant difference between vehicle‐ and Tat‐PDIA3‐treated groups, and ^‡^ *p* < .05 indicates a significant difference between control and ischemic groups). Error bars represent the standard error of the mean

In the Tat‐PDIA3‐treated control group, BDNF mRNA expression showed a significant increase of 357.2% in the hippocampal homogenates compared to that in the vehicle‐treated control group. In the vehicle‐ and Tat‐PDIA3‐treated ischemic groups, though the BDNF mRNA levels were further increased compared to that in the vehicle‐treated ischemic group, this increase in expression was not statistically significant (Figure 7).

## 4 DISCUSSION

PDIA3 is one of the molecular chaperones that reduces protein aggregates after ER stress and has a thioredoxin‐like domain and facilitates disulfide isomerase activity in denatured proteins (Hatahet & Ruddock, 2009; Kozlov et al., 2010; Määttänen, Gehring, Bergeron, & Thomas, 2010). In the present study, we focused upon the effects of exogenous PDIA3 on the proliferating cells and neuroblast numbers in the hippocampus, which is one of neurogenic regions in adult brain. First of all, we confirmed the efficient delivery of Tat‐PDIA3 in the DG by immunohistochemistry for polyhistidine because we added polyhistidine tag in the Tat‐PDIA3 vector as shown in the previous studies (Yoo et al., 2017, 2019). Tat‐PDIA3‐treated control and ischemic groups showed strong polyhistidine immunoreactivity in the DG, while vehicle‐treated control and ischemic groups did not show any prominent polyhistidine immunoreactivity in the hippocampal DG. This result suggests that PDIA3 protein is delivered into the DG and our colleagues already demonstrated the PEP‐1 fusion protein was transduced to hippocampus after ischemic insult (Cho et al., 2008).

In the present study, the administration of Tat‐PDIA3 to control gerbils did not show any significant changes in spontaneous motor activity and the number of NeuN‐immunoreactive cells in the hippocampal CA1 region. However, we observed that administration of Tat‐PDIA3 ameliorated the ischemia‐induced hyperactivity in gerbils 1 day after ischemia and significantly decreased the neuronal death and glial activation in the hippocampal CA1 region 4 days after ischemia. This result is consistent with our previous study that Tat‐PDIA3 protects neurons from ischemic damage in the gerbil hippocampus (Yoo et al., 2019).

Tat‐PDIA3 treatment significantly increased the proliferating cells and neuroblasts in the DG compared to that in the vehicle‐treated control group. These results suggest that Tat‐PDIA3 facilitates cell proliferation and neuroblast numbers in the DG of naïve gerbils. Several convincing data show that PDIA3 has regeneration activity in mechanically injured sciatic nerve (Castillo et al., 2015) and also has a role in neurite outgrowth with calcium modulation in ER (LeBlanc & Nemere, 2014). In the present study, we also observed that ischemia significantly increased the proliferating cells and neuroblasts/immature neurons in the DG 4 days postischemia/reperfusion compared to that in the vehicle‐treated control gerbils. Our colleagues previously demonstrated that transient forebrain ischemia increases the number of Ki67‐positive nuclei in the DG, while DCX‐immunoreactive neuroblasts showed similar levels in the DG at 4 days after ischemia (Choi et al., 2012). This result is consistent with previous studies that cell proliferation is significantly induced at 3–4 days after ischemia (Iwai et al., 2002; Liu, Solway, Messing, & Sharp, 1988). In contrast, DCX‐immunoreactive neuroblasts are significantly decreased at 3 days after ischemia (Pforte et al., 2005). The discrepancy may be associated with the animal models of ischemia and the treatment of vehicle (10% glycerol) in this study. In the present study, we could not observe any significant increase in cell proliferation and neuroblast differentiation in Tat‐PDIA3‐treated ischemic gerbils compared to vehicle‐treated ischemic group. One possible difference in the effects of Tat‐PDIA3 between normal and ischemic group is the oxidative damage of PDIA3 by transient forebrain ischemia since PDI family is susceptible to ROS such as lipid aldehyde (Carbone, Doorn, Kiebler, & Petersen, 2005). Another possibility is the reduction of compensatory increases of cell proliferation and neuroblasts numbers in the ischemic hippocampus because administration of Tat‐PDIA3 significantly ameliorates the neuronal damage induced by ischemia/reperfusion (Yoo et al., 2017, 2019).

Hippocampal neurogenesis is regulated by various factors including neurotrophic factors in the brain. Particularly, BDNF is most abundant and widely distributed in the brain and is closely related to neural development and plasticity (Babu, Ramirez‐Rodriguez, Fabel, Bischofberger, & Kempermann, 2009) by activating CREB and protein kinase A (Yang, Lin, Chuang, Bohr, & Mattson, 2014). In the present study, we observed the BDNF mRNA levels in the hippocampal homogenates and found significant increases after Tat‐PDIA3 treatment in control gerbils. In addition, transient forebrain ischemia drastically increased BDNF mRNA expression levels in the hippocampus of vehicle‐ and Tat‐PDIA3‐treated groups. This result is consistent with a previous study that BDNF mRNA levels were increased 3 or 7 days after ischemia compared to sham‐ischemic group (Prosser‐Loose, Verge, Cayabyab, & Paterson, 2010). However, we could not observe any significant difference in BDNF mRNA levels between vehicle‐ and Tat‐PDIA3‐treated groups 4 days after ischemia/reperfusion. Similarly, the number of pCREB‐immunoreactive nuclei was significantly increased in the Tat‐PDIA3‐treated control gerbils compared to that in the vehicle‐treated control groups. In addition, transient forebrain ischemia significantly increased the number of pCREB‐immunoreactive nuclei compared to that in the control. However, administration of Tat‐PDIA3 did not show any significant changes in the number of pCREB‐immunoreactive nuclei 4 days after ischemia/reperfusion compared to that in the vehicle‐treated ischemic group. These results suggest that Tat‐PDIA3 promotes the cell proliferation and neuroblast differentiation and these effects may be closely related to the upregulation of BDNF mRNA levels and phosphorylation of CREB in the naïve gerbils, but not in the ischemic gerbils. PDIA3 is associated with fluoxetine, an antidepressant, since administration of fluoxetine increases PDIA3 levels in the hippocampal homogenates (Plaingam, Sangsuthum, Angkhasirisap, & Tencomnao, 2017). Oral administration of fluoxetine, an antidepressant, increases large‐sized perforant path‐granule cell synapse (Kitahara et al., 2016) and also the expression of BDNF, in gerbils, after transient forebrain ischemia (Kim et al., 2007) and hippocampal neurogenesis (Encinas, Vaahtokari, & Enikolopov, 2006; Santarelli et al., 2003). However, fluoxetine has no effect on ischemia‐induced increase in neurogenesis in the rat DG (Choi, Cho, & Kim, 2007). This result coincides with our study that Tat‐PDIA3 has no effect on neurogenesis in the ischemic gerbils.

There have been conflicting evidences about the source of new neurons in the CA1 region after ischemia and potentials to regenerate in the DG. A study demonstrates the source of new neurons in the hippocampal CA1 region comes from DG (Bendel et al., 2005), while other study shows limited information of their origins (Nemirovich‐Danchenko & Khodanovich, 2019) because of low ability for self‐renewal (Mignone, Peunova, & Enikolopov, 2016). In the SVZ of lateral ventricle, only 20%–30% cells are divided symmetrically and have self‐renewal activity in the niche for several months before generating neurons (Obernier et al., 2018), while asymmetric divisions are dominantly found in the progenitor cells of the DG (Encinas et al., 2011). However, in the present study, we could not elucidate the correlation between enhanced neurogenesis in the DG and regeneration in the hippocampal CA1 region and it needs to be elucidated.

In conclusion, our results show that Tat‐PDIA3 enhances cell proliferation and neuroblast numbers in the DG and these effects may be associated with upregulation of BDNF mRNA expression and increases in the phosphorylation of CREB in the hippocampus. However, Tat‐PDIA3 administration fails to affect the ischemia‐induced cell proliferation and neuroblast numbers in the DG with minimal change in BDNF mRNA expression and phosphorylation of CREB.

## CONFLICTS OF INTEREST

None declared.

## AUTHOR CONTRIBUTION

DYY, SBC, SYC, and IKH designed the study and wrote the manuscript. DYY, HYJ, SK, SMN, and JWK conducted the in vivo experiments. SBC and DWK made Tat‐PDIA3 fusion protein and confirmed the successful expression of PDIA3 protein. SMM, YSY, and DWK participated in the design of experiment and edited the manuscript.

## Data Availability

The data that support the findings of this study are available from the corresponding author upon reasonable request.
